# Dense neural network outperforms other machine learning models for scaling-up lichen cover maps in Eastern Canada

**DOI:** 10.1371/journal.pone.0292839

**Published:** 2023-11-20

**Authors:** Galen Richardson, Anders Knudby, Wenjun Chen, Michael Sawada, Julie Lovitt, Liming He, Leila Yousefizadeh Naeni

**Affiliations:** 1 Department of Geography, Environment and Geomatics, University of Ottawa, Ottawa, Ontario, Canada; 2 Canada Centre for Mapping and Earth Observation, Natural Resources Canada, Ottawa, Ontario, Canada; 3 Département de Géomatique Appliquée, Université de Sherbrooke, Sherbrooke, Québec, Canada; Universidade Federal de Uberlandia, BRAZIL

## Abstract

Lichen mapping is vital for caribou management plans and sustainable land conservation. Previous studies have used random forest, dense neural network, and convolutional neural network models for mapping lichen coverage. However, to date, it is not clear how these models rank in this task. In this study, these machine learning models were evaluated on their ability to predict lichen percent coverage in Sentinel-2 imagery in Québec and Labrador, Canada. The models were trained on 10-m resolution lichen coverage (%) maps created from 20 drone surveys collected in July 2019 and 2022. The dense neural network achieved a higher accuracy than the other two, with a reported mean absolute error of 5.2% and an R^2^ of 0.76. By comparison, the random forest model returned a mean absolute error of 5.5% (R^2^: 0.74) and the convolutional neural network had a mean absolute error of 5.3% (R^2^: 0.74). A regional lichen map was created using the trained dense neural network and a Sentinel-2 imagery mosaic. There was greater uncertainty on land covers that the model was not exposed to in training, such as mines and deep lakes. While the dense neural network requires more computational effort to train than a random forest model, the 5.9% performance gain in the test pixel comparison renders it the most suitable for lichen mapping. This study represents progress toward determining the appropriate methodology for generating accurate lichen maps from satellite imagery for caribou conservation and sustainable land management.

## 1. Introduction

In 2016, *Rangifer tarandus*, commonly known as the caribou, was categorized as a vulnerable species due to an observed decline of 40% between the 1990s and 2015 [1]. Caribou populations are threatened by changes to their habitat, unregulated hunting, and herd fragmentation due to human-made infrastructure [1–3]. Terricolous (ground-dwelling) lichen is a primary food source for caribou, comprising 75% and 25% of their winter and summer diets respectively [2]. In recent decades, ecological and climate changes in the caribou habitat, such as the expansion of shrublands and managed forests, have reduced the abundance of lichen in caribou ranges [4–6]. The increasing frequency, pollution, severity, and spatial extent of forest fires in the boreal region have also impacted the availability of lichen, as lichen requires decades to fully recover [3–5, 7]. Mapping and monitoring lichen availability are thus critical for informing sustainable land management policy and caribou recovery plans.

The base data for lichen mapping generally consists of field measurements, digital photographs, unmanned aerial vehicle (UAV) surveys, and satellite imagery. These data are used to train models to detect lichen at different spatial resolutions. Early lichen mapping models focused primarily on linking the spectral characteristics of lichen to remotely sensed imagery [8–10]. For example, the normalized difference vegetation index (NDVI) was used to detect lichen in Landsat TM imagery located in tundra regions with no tree canopy coverage [9]. However, many caribou ranges exist below the tree line, and models based on NDVI struggle to distinguish lichen from other vegetation in regions with canopy cover [2]. To address this issue, Théau et al. improved the classification of lichen in Landsat TM imagery by implementing Spectral Mixture Analysis [2]. This approach was able to correctly detect lichen in 75% of the 24 field-verified sites but tended to overestimate lichen coverage (%) when compared to lichen maps from aerial surveys [2].

A wide range of studies have broadly found that machine learning models for classification tend to produce higher accuracy compared to traditional parametric models [11]. To generate sufficient training data for machine learning models, researchers have implemented multi-scaled approaches to model development. This involves training models on higher-resolution data and then applying them to lower-resolution data to make predictions (maps) over large areas. This technique has been used to map tree mortality [12], detect invasive plant species [13], and for lichen mapping [4, 14, 15].

One of the most common machine learning models implemented in multi-scaled lichen mapping is random forest [16]. Developed by Breiman, random forest is a non-parametric ensemble classifier consisting of a collection of decision trees that are each created from random subsets of training data, with model predictions based on the average/majority of individual tree predictions [16–18]. The user can define the number of trees included in the model, however each decision tree within the random forest operates independently [17]. The advantages of random forest models are their ease of optimization and high predictive performance [11, 16, 18, 19]. Random forest models have been successfully implemented in multi-scale lichen mapping applications [4, 15]. For example, MacAnder et al. developed a methodology to classify lichen in Landsat 7 imagery, using a random forest model and resampled UAV orthomosaics as training data, with an R^2^ of 0.71 and a Mean Average Error (MAE) of 5.2% in interior Alaska and Yukon datasets [4]. According to the authors of this study, the model tended to overestimate lichen coverage in areas with less lichen and underestimate lichen coverage in areas with high amounts of lichen [4]. Other research has shown that estimating lichen coverage through direct scaling of UAV orthomosaics to Landsat imagery is associated with high uncertainties due to poor co-registration between datasets of significantly different spatial resolutions [20]. Therefore, extra care must be taken to minimize errors arising from co-registration issues.

Recent approaches to lichen mapping involve using neural networks, to create lichen maps. Neural networks can be designed to have high predictive performance at regression and classification tasks, but are substantially less interpretable, slower to train, and harder to optimize than random forest models [11, 19, 21]. For example, He et al. used binary lichen maps from WorldView (WV) imagery (50 cm) to train a regression dense neural network to predict lichen coverage (%) in Landsat 8 OLI imagery (30 m) [20]. This model had an R^2^ value of 0.60 and an MAE of 6.6% when comparing the lichen cover from WV imagery to the lichen cover in Landsat 8. The training data in that study used binary lichen maps at the 50 cm resolution, and it was reliant on at-cost WV imagery [20]. Another type of model that has been used for lichen mapping is Convolutional Neural Networks (CNNs). Unlike single-pixel classifiers, CNNs can create inferences based on image texture, intensity, and other spatial patterns, and use these along with spectral information to classify pixels in an image [22, 23]. CNNs have many different remote sensing applications, such as detecting trees and buildings [24, 25], segmenting ecotypes [22], and detecting landcover change [26]. Jozdani et al. and Richardson et al. have explored the use of CNNs for lichen mapping with high resolution imagery [27, 28]. Jozdani et al. implemented a CNN for binary classification of lichen in WV imagery (50 cm), with an overall accuracy of 85.28% and an F1-score of 84.38%, outperforming a random forest classifier [27]. Richardson et al. developed UAV LiCNN, a multi-scaled CNN trained on vegetation plot photos to classify pixels in UAV orthomosaics (2 cm) [28]. This model had mean user and producer accuracies of 85.84% and 92.93% respectively when classifying pixels containing a high lichen percentage but had lower accuracies at classifying lichen in shadows [28].

A small body of published work has focused on implementing CNNs for regression applications. Regression CNN models have been implemented to map freshwater shortages, bathymetry, chlorophyll-a (Chl-a) in lakes, and lichen [19, 29–32]. Aptoula et al. developed a six-layer CNN which used 3x3 tiles with 12 Sentinel-2 bands to estimate Chl-a in Lake Balik [32]. The authors discovered that CNNs outperformed non-deep learning methods and that a two-dimensional CNN outperformed a three-dimensional CNN [32]. Erlandsson et al. developed a Landsat-based lichen biomass model that used Scandinavian field records collected over twenty years to inform a deep CNN [29]. This model had an R^2^ of 0.57, and an MAE of 7.5% when predicting lichen volumes. Erlandsson et al. reported that the design of the datasets was likely unable to compensate for spatial autocorrelation and that the results are suspected to be overly optimistic [29]. The low number of training samples (3,290) and complex structure of this model may also indicate an overdetermined CNN, further supporting the authors’ conclusion.

An issue for many lichen mapping models is the availability of transferable true observations to open-source satellite data. Previous studies have created fractional lichen coverage maps using Landsat imagery due to the vast archive of imagery available [2, 4, 9, 15, 20, 33]. However, with a pixel resolution of 30 m, it is challenging to align Landsat imagery with high resolution datasets. Sentinel-2 imagery is freely available, has a higher resolution of 10 m in some bands for more accurate alignment to UAV datasets, and could serve as an intermediate scaling-up step to Landsat datasets. Additionally, Sentinel-2 imagery can be used to create current lichen maps with a higher spatial resolution than Landsat imagery.

Previous research has shown that random forest models, dense neural networks, and deep CNNs are all effective machine learning models which have been used for lichen mapping. However, to date, it is unclear which type of model performs best when compared on the same dataset. In this present study, these models were assessed in a study region with a dominant lichen species of *Cladonia stellaris*, consisting of the Manicouagan Caribou Range and the Red Wine Mountain Caribou Range in Québec and Labrador Canada. This study assesses (1) which regression machine learning methodology is most effective at predicting lichen coverage (%) and (2) whether Sentinel-2 imagery can be used for regional lichen mapping. The results serve as guidance for future lichen mapping efforts and other landcover mapping applications.

## 2. Materials and methods

### 2.1 Lichen map datasets

**Fig 1:**
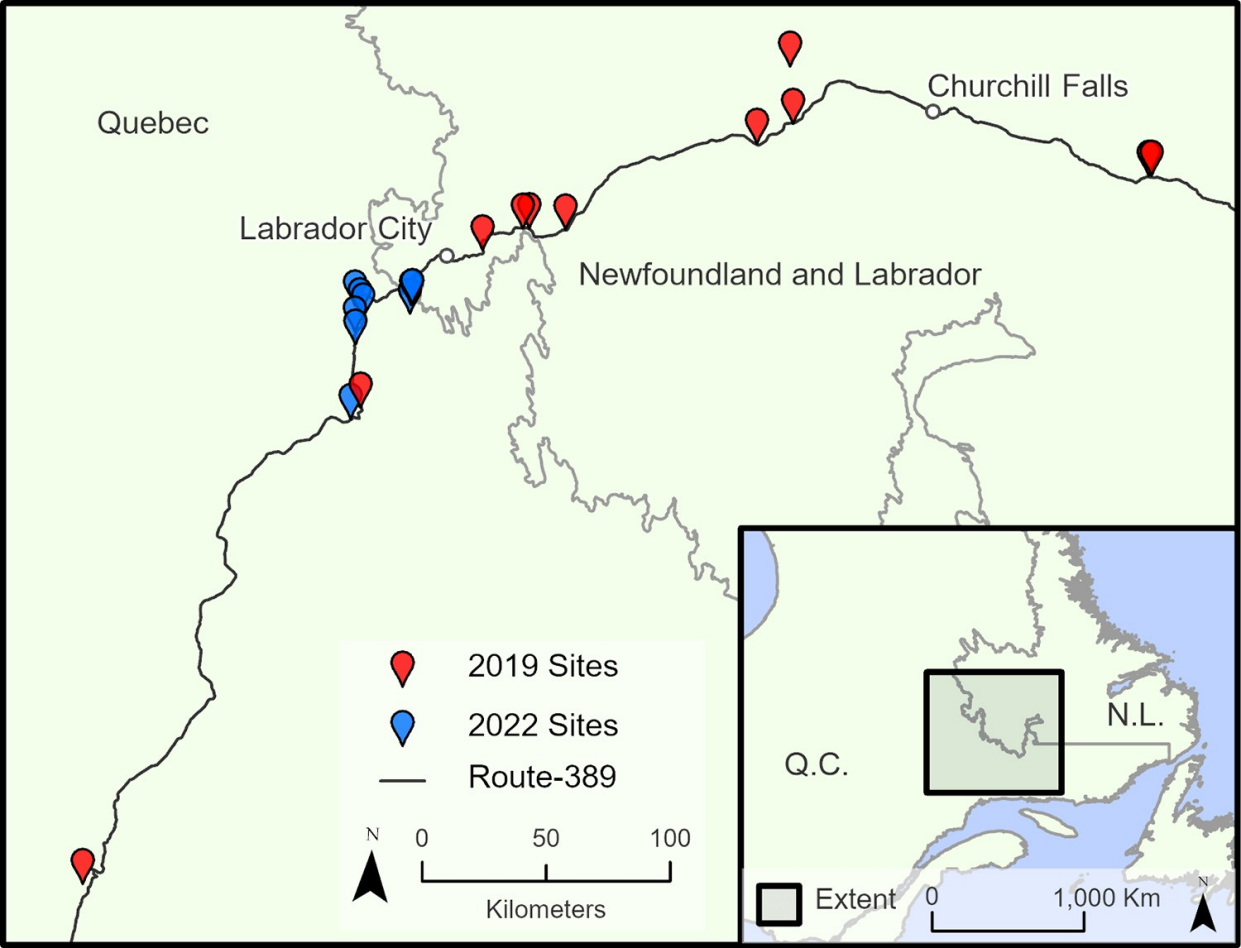
Map of the sites visited in this study. Contains information licensed under the Open Government Licence–Canada.

The lichen maps used for model training and evaluation were created from 20 orthomosaics derived from UAV imagery collected between July 24—July 31, 2019, and June 25—July 2, 2022. The imagery was collected at sites located along the Québec Route-389 or the Trans-Labrador Highway and were accessible by foot or vehicle (Fig 1). The sites represented a variety of different land covers, topographies, and a large selection of different lichen conditions in the Québec and Labrador region. All sites were included in this study to maximize the different land cover conditions the machine learning models were exposed to.

The 2019 UAV imagery was collected using either a DJI Inspire 1 with a Sentra Double 4 K Normalized Difference Red Edge camera or a DJI Mavic 2 pro with its stock RGB camera. One frame per second was extracted from the 4 K video for the DJI Mavic 2 Pro, while one photograph per second was captured by the Sentra Double 4 K camera, following the methodologies outlined by Fernandes et al. and Leblanc [34, 35]. One of the 2019 UAV image datasets was flown with a DJI Mavic 2 Pro and captured images with a 1 cm resolution over an area of 2.5 ha. The 11 other sites visited in 2019 had a spatial resolution of 2 cm resolution and areas of approximately 17 ha. The 2022 UAV imagery consisted of eight sites and was collected using a DJI Phantom 4 Pro with its stock RGB camera. One photograph was collected every two seconds during operation and all resulting orthomosaics had a 1.5 cm resolution over areas of approximately 10 ha.

Pix4D mapper version 4.4.12 and 4.75 was used to process the 2019 and 2022 UAV orthomosaics respectively. All UAV orthomosaics used a minimum of three high-accuracy Global Navigation Satellite System (GNSS) data ground control points to improve the locational accuracy of the UAV orthomosaics.

**Fig 2:**
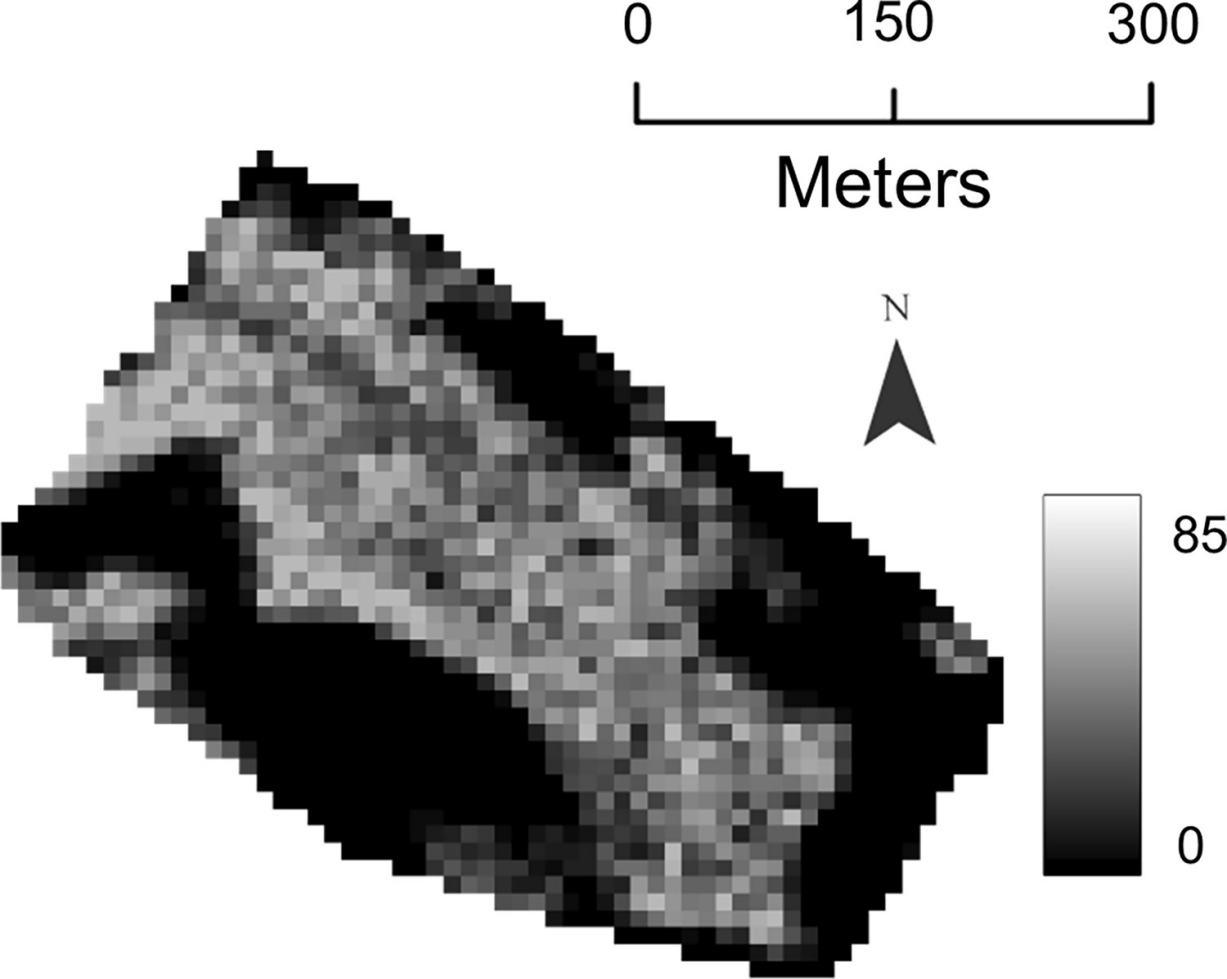
Example of a 10-m lichen map.

The 1 cm and 1.5 cm resolution orthomosaics were resampled to 2 cm pixels with bilinear resampling before classification. UAV-LiCNN, a methodology developed by Richardson et al., was used to classify lichen in the orthomosaics into three classes, which represented 0% lichen coverage, 50% lichen coverage, and 100% lichen coverage [28]. The classification outputs were manually cleaned using the pixel editor in ArcGIS Pro to correct substantial misclassifications of shadows, bright logs, and sand. Lichen coverage (%) maps at 10 m resolution were generated from the UAV lichen maps by using the block statistics tool in ArcGIS Pro (Fig 2). The mean lichen coverage (%) from the UAV lichen maps was calculated for each Sentinel-2 pixel, creating 10-m lichen maps where pixel values ranged from 0 to 100.

### 2.2 Sentinel-2 imagery

The Sentinel-2 surface reflectance imagery used in this study was from the Harmonized Sentinel-2 MSI: Multispectral Instrument, Level-2A collection accessed through Google Earth Engine (GEE). The harmonized surface reflectance collection was selected to reduce pre-processing and to ensure that the DN values in the 2022 imagery were in the same range as those from previous years. Sentinel-2 images that did not contain clouds or cloud shadows over any of the sites visited that year were selected for this study. The Sentinel-2 imagery used in this study was captured on August 26, 2019, and August 18, 2022 respectively. The imagery was downloaded from GEE, with each band at a resolution of 10 m in a GeoTIFF format.

**Fig 3:**
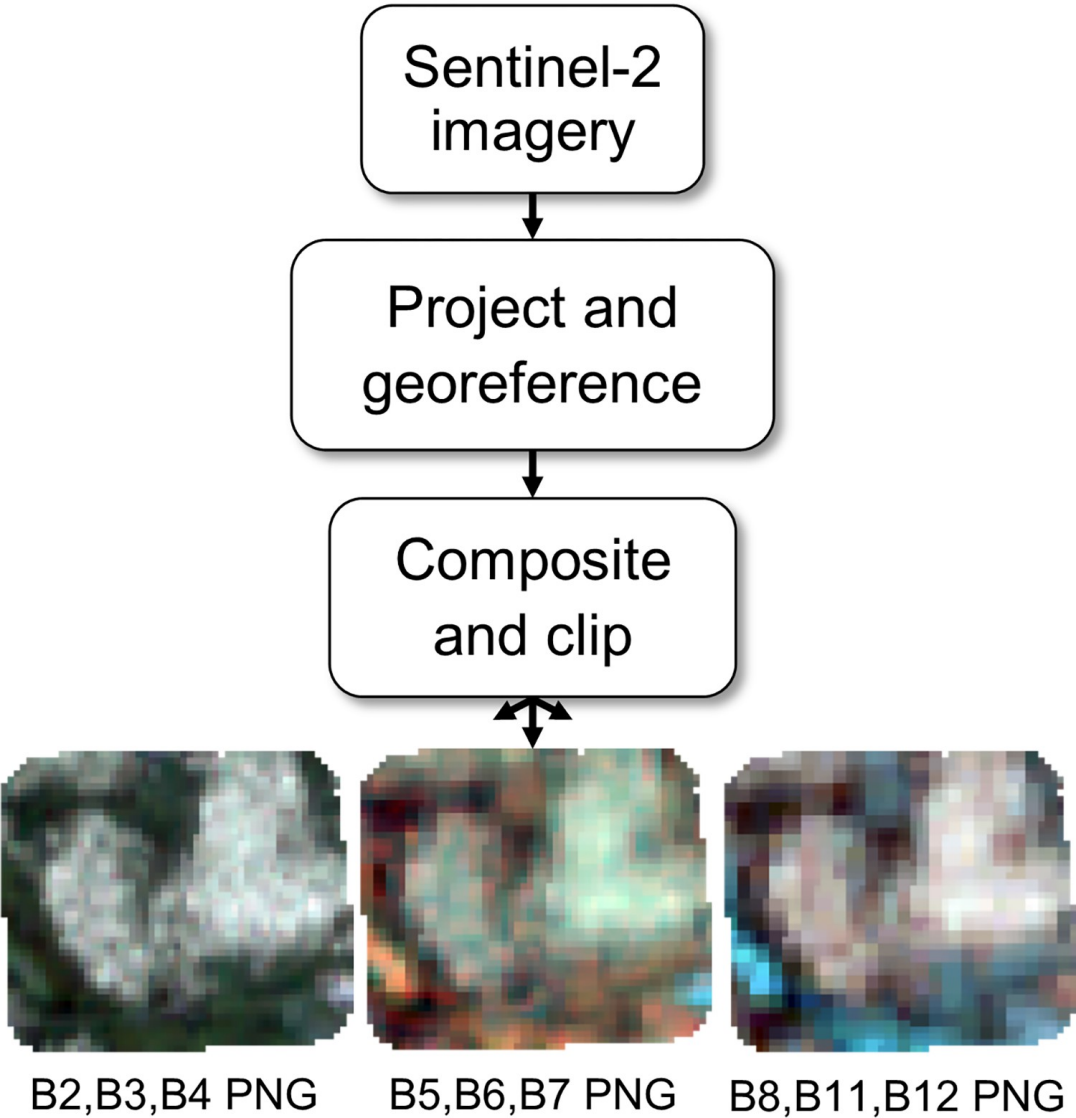
Sentinel-2 imagery processing workflow.

For this study we excluded all natively 60-m bands due to their low spatial resolution. Band 8A (20 m) was also excluded due to the spectral closeness with Band 8 (10 m) and lower spatial resolution. Fig 3 outlines the workflow for processing the Sentinel-2 imagery. The remaining nine bands were composited and reprojected to their respective UTM projection with bilinear resampling. The 12.5 m geolocation accuracy of Sentinel-2 required the imagery to be georeferenced to the UAV orthomosaics [36]. This was done in ArcGIS Pro with one tie point to translate Sentinel-2 imagery into alignment. Then the Sentinel-2 imagery was clipped and saved as three 16-bit portable network graphic (PNG) files for further processing in TensorFlow.

### 2.3 Dataset preparation

**Fig 4:**
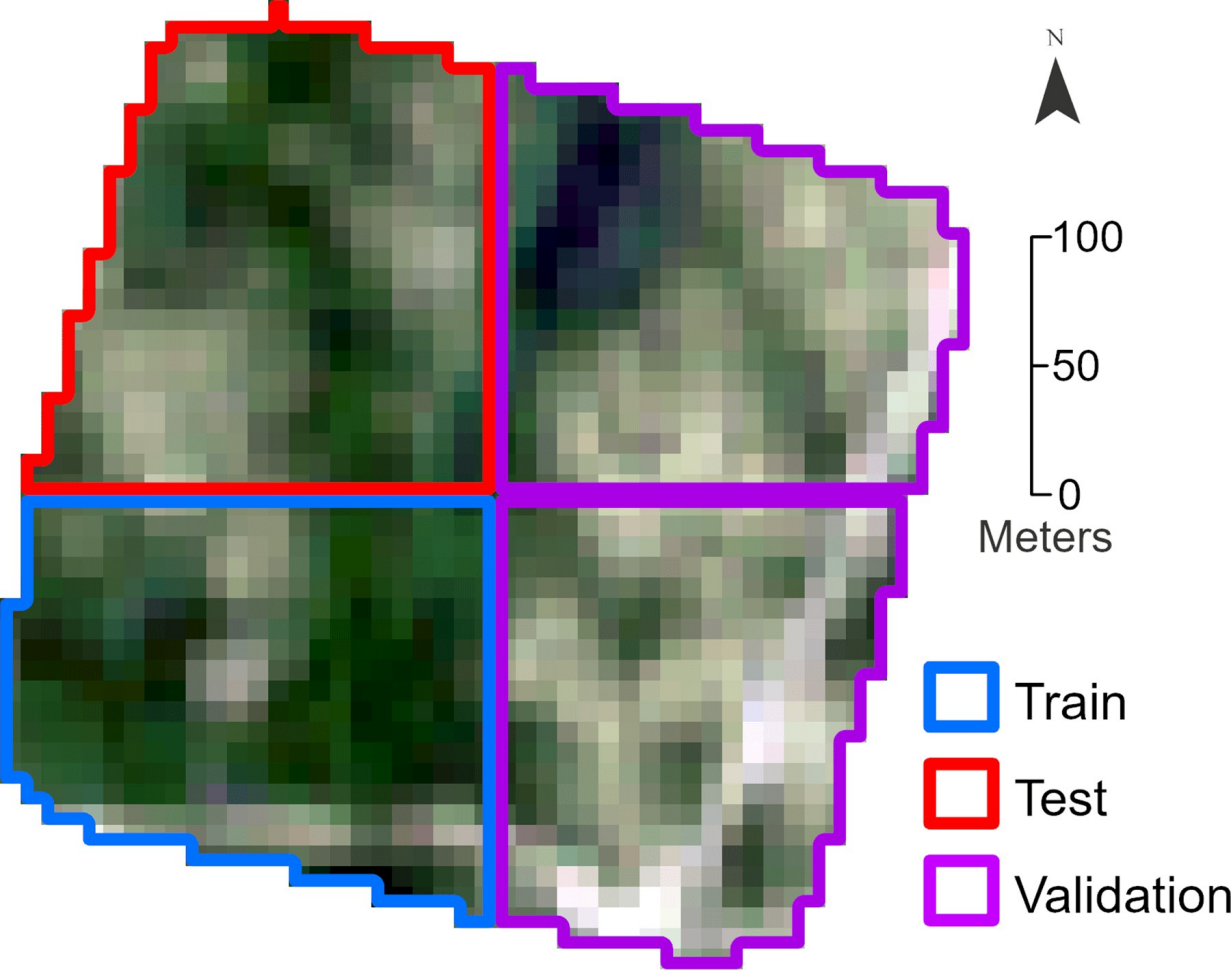
Example of quadrant blocking used to minimize spatial autocorrelation between the datasets.

To create a training dataset that exposed the machine learning models to the diversity of landscapes found in the UAV orthomosaics, portions of all sites, excluding the smaller 2.5 ha site, were incorporated into the train, validation, and test datasets. An important consideration was the spatial autocorrelation found in the Sentinel-2 imagery and 10-m lichen map. Previous studies that have presented seemingly outstanding performances of CNNs commonly use random cross-validation, which does not account for spatial autocorrelation between training and testing datasets [37]. However, mapping studies that do not address spatial autocorrelation can have overoptimistic assessments of model predictive power [37–39]. To avoid this, we implemented spatial partitioning (also known as “blocking”), where we split the sites into quadrant blocks, assigning blocks rather than individual pixels to the train validation, and test datasets (Fig 4). Following guidance from Ploton et al., this strategy produced sufficiently large blocks to address spatial autocorrelation within each of the orthomosaics by largely eliminating pseudoreplicate issues and the interdependence between train, validation and test data that would ensue with a random sampling of spatially autocorrelated data [38]. However, at the same time, the model is still exposed to many different landscapes. All orthomosaics were subjected to blocking except for the small 2.5 ha site which was completely included in the test dataset.

The Sentinel-2 imagery and 10-m lichen maps were clipped to the quadrant block extents and split with 60%, 20%, and 20% of the blocks allocated to the train, validation, and test datasets respectively. We initially tested randomized allocation of blocks to the train, validation, and test datasets, but this resulted in uneven distribution of different landscapes and lichen percent coverages. Additionally, we wanted to ensure that the test and validation datasets contained data from as many different sites as possible. To address these issues, we manually allocated the blocks to ensure that each dataset had a balanced representation of different landscapes and lichen coverages. Lichen near sand, recent forest fire burns, lichen regrowth, high and low lichen coverage patches, roads, lichen in forests, lichen in wetlands, lichen surrounding downed trees, and rocks with lichen were some of the landscapes considered when deciding which block went into which dataset. As an additional step, before proceeding we verified that the datasets had similar lichen percent coverage histogram distributions.

### 2.4 Convolutional neural network dataset preparation

**Fig 5:**
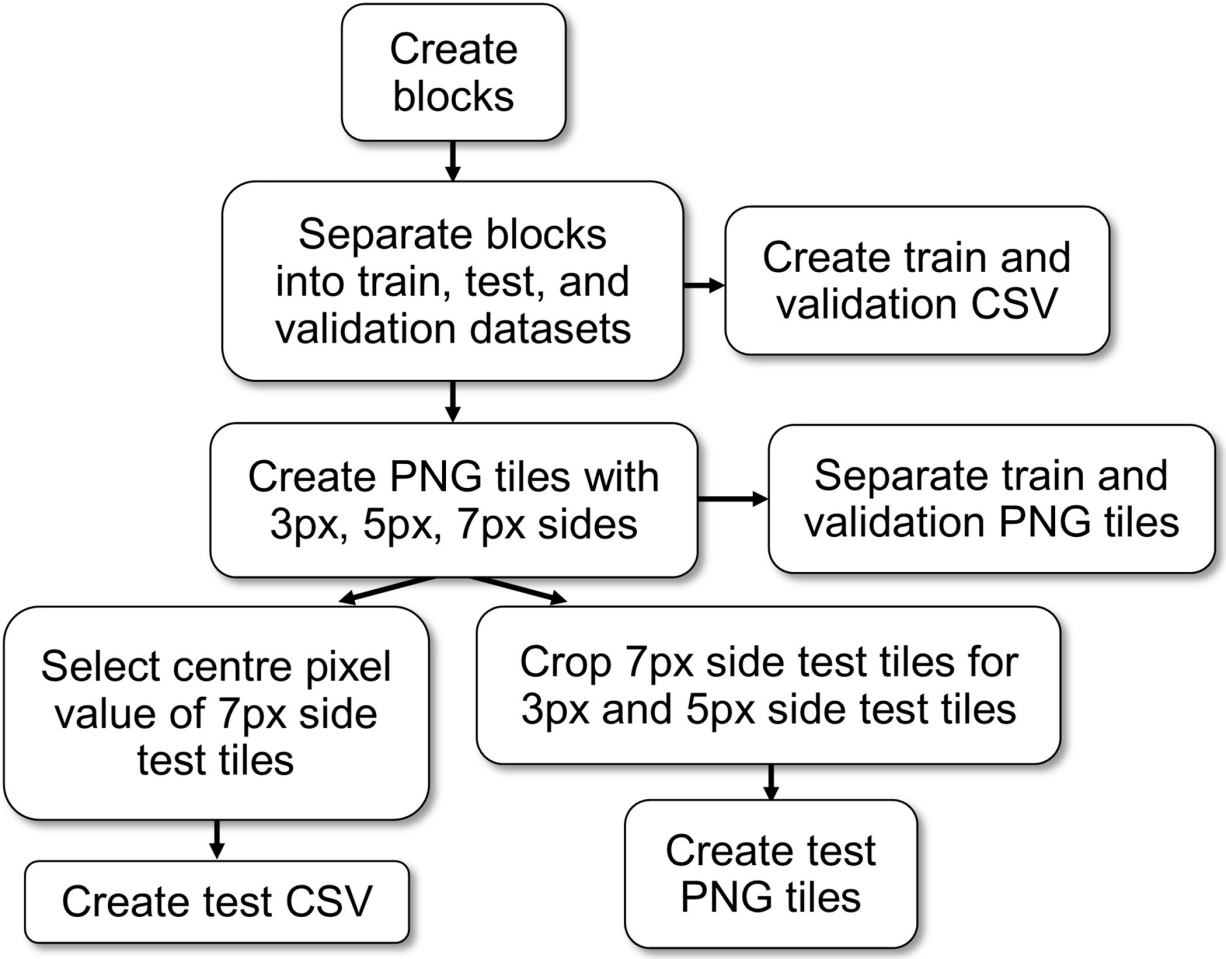
Workflow depicting dataset preparation for the CNN, dense neural network, and random forest models.

The inputs for the CNN model were square tiles generated from Sentinel-2 imagery blocks, and the output was a single value which represented the lichen percent coverage of the centre pixel of each tile. PNG files were used as a file format in this study for CNN training as TensorFlow does not yet offer native support for Tag Image File Format (TIFF) files with more than three bands. We tested neural network training with 9-band TIFF files in TensorFlow using the *tifffile* python package and found that it was significantly slower than loading three PNG containing the same data. The quadrant blocks designated for each dataset were iterated through, and tiles with sides of 3 pixels (px), 5 px, and 7 px were generated from the three Sentinel-2 PNG files and the 10-m lichen maps (Fig 5). There was overlap between each tile in their respective dataset to maximize the number of unique training inputs.

Datasets with larger tile sides had fewer possible tiles created within each block since all tiles containing NoData values were excluded from the datasets. The number of training and validation inputs differed with the tile size, with 11,621 training inputs for the 7 px tiles and 19,594 training inputs for the 3 px tiles. To ensure consistent test datasets for comparing models, only the central values of the 7 px tiles were used. We cropped the 7 px test dataset tiles to generate 3 px and 5 px tiles for their respective test datasets. This resulted in 3,963 test pixels used to compare the different models in this study.

### 2.5 Random forest and dense neural network dataset preparation

For each site, the pixel values within the three Sentinel-2 PNG files and the 10-m lichen map quadrant blocks were read and extracted into a comma-separated value (CSV) file for the random forest and dense neural network (Fig 5). The training dataset and validation dataset consisted of 24,998 and 7,794 pixels respectively. To ensure consistency in model comparison, we limited the test dataset for the random forest and dense neural network to only contain the central pixel also found in the CNN test dataset, resulting in 3,963 test pixels.

### 2.6 Random forest training

The random forest model was created using the Sci-kit Learn random forest regressor [40]. The training and validation datasets were used to optimize model hyperparameters *n_estimators* (number of trees) and *max_features* (number of subsets to consider in node splitting). Model performance was assessed with MAE because the average performance of these models over large regions was the main measure of model efficacy.

We tested values of 30, 100, 300, and 1000 for *n_estimators*, and auto, sqrt, and log2 for the *max_features* parameters. The model that performed the best on the validation dataset had *n_estimators* = 1000 and *max_features* = log2, taking 5 minutes to train on an Intel i5-12600k CPU. The best performing model had an R^2^ value of 0.73, and an MAE of 5.5% when compared to the validation dataset.

### 2.7 Neural network parameter and model selection

For the dense neural network and CNN models, variations of dense, drop out, batch normalization, and one-dimension convolutional layers were tested in different arrangements. Various forms of CNN model architecture were tested including combinations of 2D convolutions, cropping, concatenations, and flattening layers. The Adam optimizer and a combination of rectified linear unit (ReLU) and linear activation functions were used in our final models. S1 Appendix outlines our choice of activation functions, loss functions, and optimizers in model selection.

Hyperparameter tuning was completed as an iterative process using a custom Python script (v3.8) that cycled through various settings and logged results. We then evaluated the loss curves of the best performing models to determine if they showed signs of overfitting. Overfitting is seen when the model does not generalize well from the training data to the validation data [41–43]. Neural networks are prone to overfitting due to the large number of parameters that can learn to map inputs to outputs without being able to generalize beyond the training dataset [41, 43]. To avoid selecting a model that has overfit, we implemented an early stopping function. We also disregarded models which developed significant gaps between the training and validation loss curves, because that is a sign of learned noise patterns in the training dataset [41, 43]. Models which had the lowest MAE on the validation dataset and did not show signs of overfitting were evaluated in this study.

### 2.8 Dense neural network training

**Fig 6:**
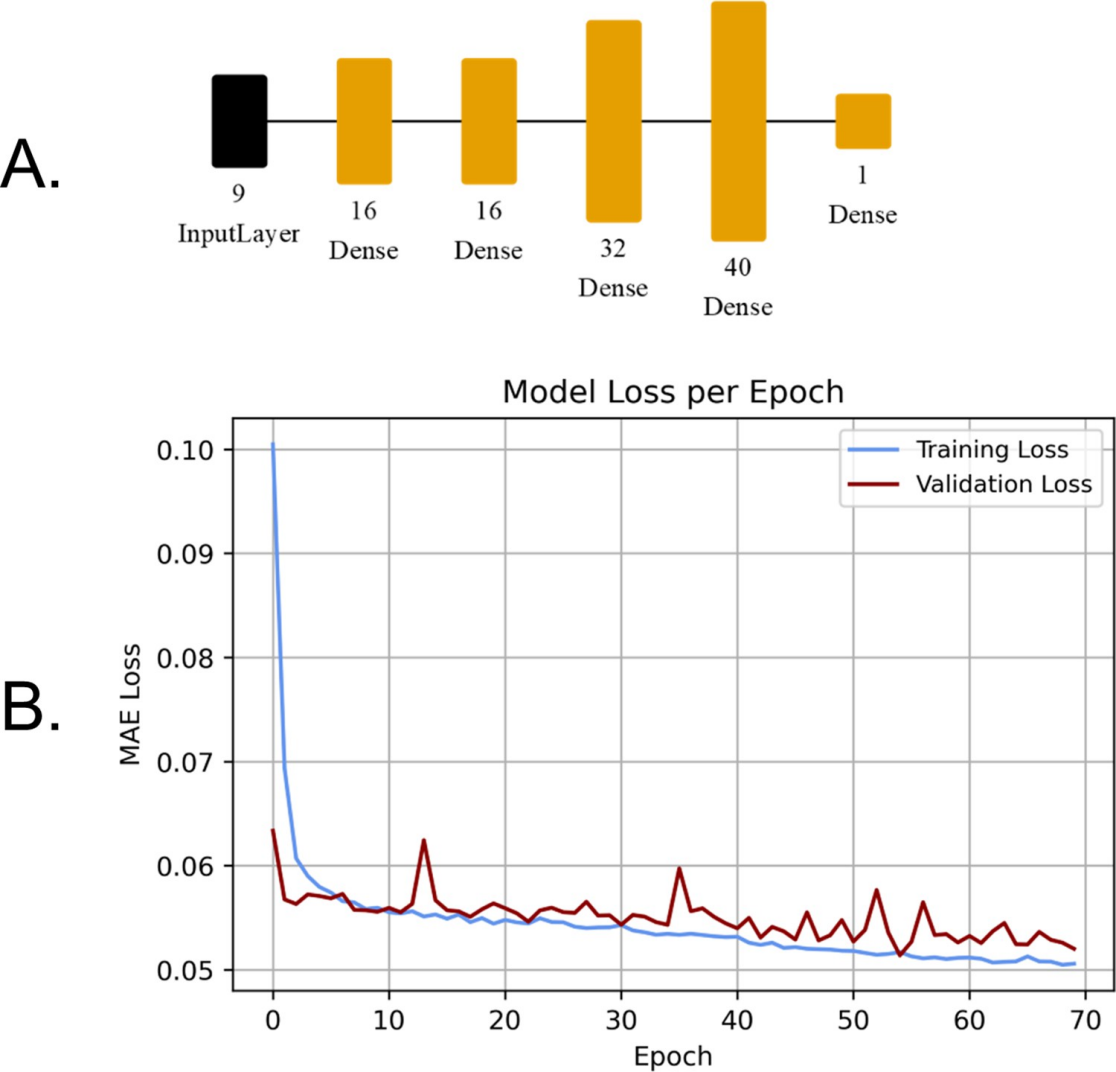
A. Visual representation of the dense neural network model using Net2Vis [44], B. training and validation loss curves.

The dense neural networks assessed in this study were created in TensorFlow 2.8 and consisted of dense layers and input layers. The dense neural network design started from the three-layer model He et al. proposed, which was improved upon by adding layers and altering parameters [20]. The input layer consisted of a tensor containing the nine Sentinel-2 band values, and the output value was the estimated lichen percent coverage of the input pixel. The architecture of the model that performed the best consisted of four dense layers that used a Rectified Linear Unit (ReLU) activation function, while the last dense layer had a linear activation function. This model contained 2,337 trainable parameters and was compiled with a learning rate of 0.001 using the Adam optimizer. It had a batch size of 1000 with 225 steps per epoch and took 66 seconds to train on an Nvidia RTX 3070ti graphics card. The model had a patience of 15 epochs; if the validation loss did not decrease over this time the best model weights were saved, and the training would end. The model weights were saved at epoch 54 since the validation loss did not improve after that point (Fig 6). The model that performed the best had an R^2^ of 0.76, and an MAE of 5.1% when compared to the validation dataset.

### 2.9 Convolutional neural network training

The objective for the CNN models was to interpret a tile of Sentinel-2 imagery and make an estimate of the lichen percent coverage of the centre pixel. The training tiles were passed through a data augmentation step, which randomly flipped and rotated tiles to expose the model to different orientations. To reduce the number of features, the CNNs in this study use valid padding strategies to discard the data at the edges of the tile [19, 45]. Valid padding ensures that convolutional filters are only applied to values in the tile and that the height and width of the output tile is eroded [19, 45, 46]. Same padding maintains the same output tile size as the input by adding zeros to the edges and is used to maintain the size of the feature map while increasing the complexity of the model [45, 46]. We created a CNN that used same padding to increase the complexity of the feature map and valid padding to erode the tile until the feature map resembled a shape of 1x1 with the number of bands equal to the number of filters used. At this point, the feature map was flattened and passed through a series of dense layers that returned a single percent lichen coverage prediction.

**Fig 7:**
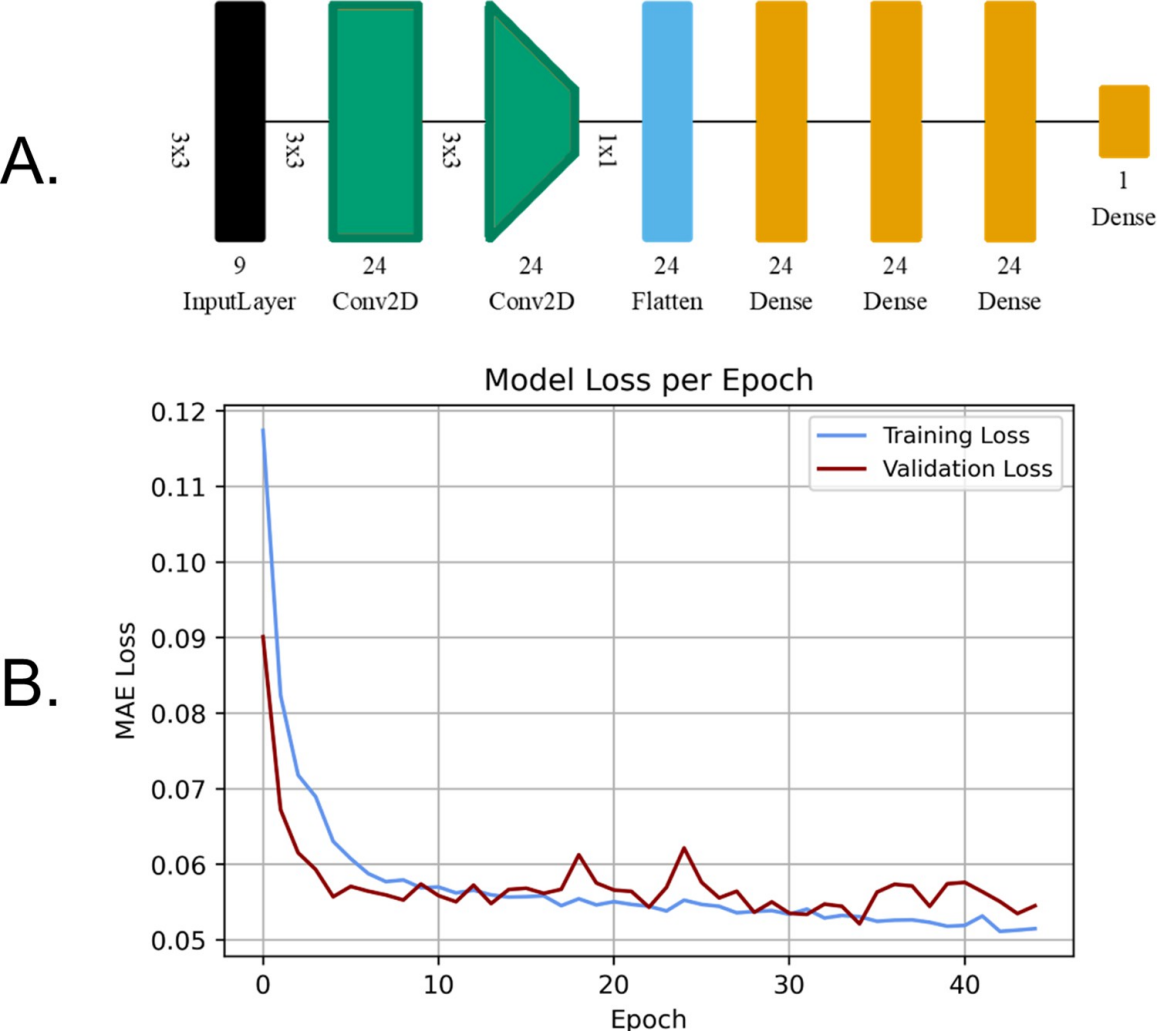
A. Visual representation of the CNN model using Net2Vis [44], B. training and validation loss curves.

The CNN that performed the best in this study was created in TensorFlow 2.8 and consisted of two-dimension convolutional, flatten, and dense layers. The two-dimension convolutional layers and the first three dense layers of this model used a ReLU activation function, and the last dense layer used a linear activation function. The model contained 9,001 trainable parameters and was compiled with a learning rate of 0.001 using the Adam optimizer. It had a batch size of 500 with 80 steps per epoch and took 13 minutes to train on an Nvidia RTX 3070ti graphics card. The model had a patience of 10 epochs and the weights were saved at epoch 34 since the validation loss did not continue to decrease after that point (Fig 7). The CNN that performed the best had an R^2^ of 0.75, and an MAE of 5.2% when compared to the validation dataset.

## 3. Results

### 3.1 Test dataset pixel comparison

**Fig 8:**
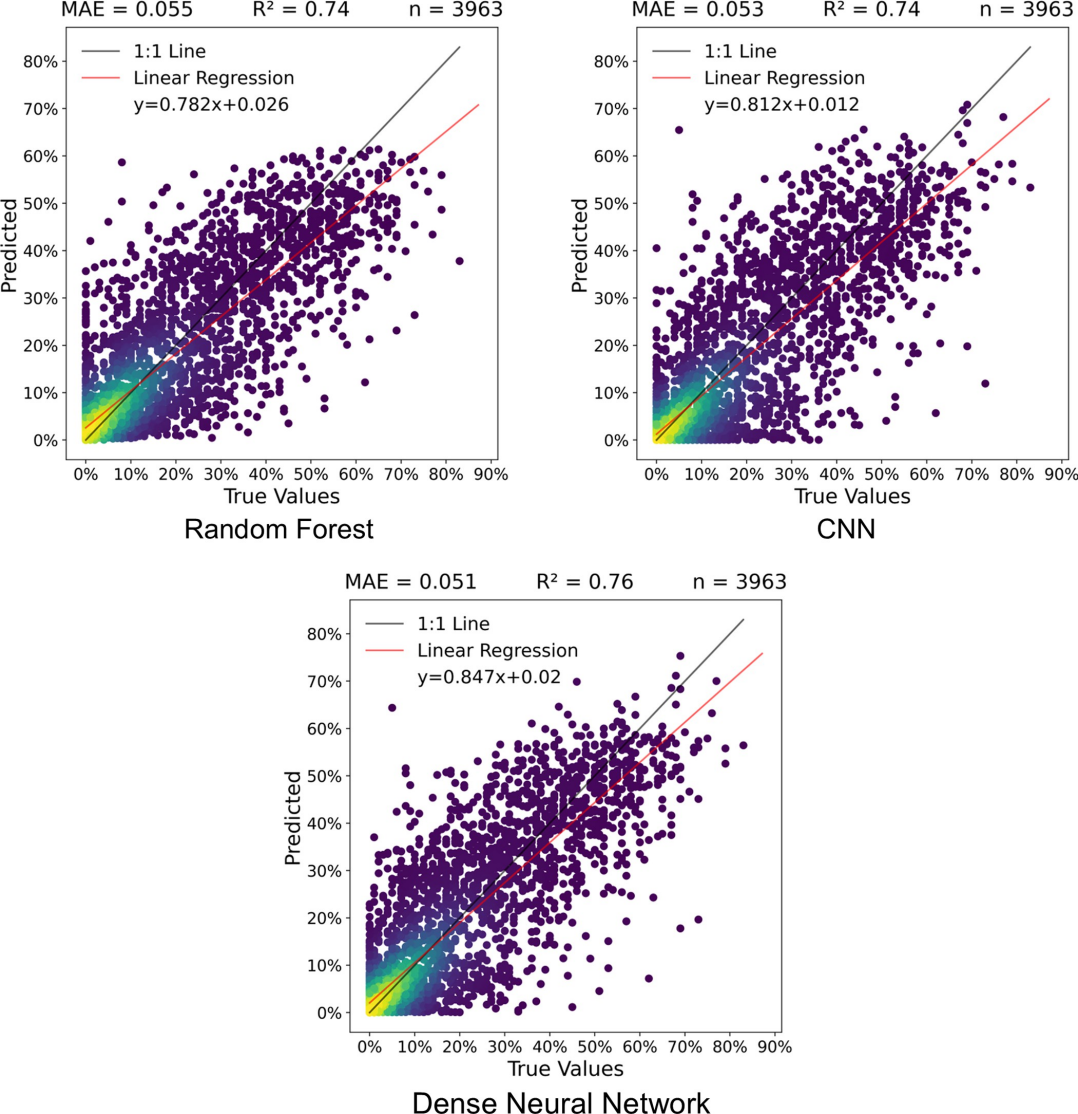
Pixel lichen percent coverage scatterplots, MAE, and R^2^ values for the model performance on the test dataset in this study. Brighter yellow colours indicate a higher frequency of points.

The dense neural network outperformed the random forest and CNN models in the test dataset pixel comparison, with an R^2^ value of 0.76 and an MAE of 5.2% (Fig 8). The random forest and CNN had MAEs of 5.5% and 5.3% respectively, and each had R^2^ values of 0.74. All three models tended to under-predict pixels with high lichen coverage. The dense neural network and random forest model had similar RMSE values of 0.089 and 0.088 respectively, while the CNN had a higher RMSE of 0.093. The random forest model seemed to misclassify more non-lichen pixels (true value of zero) as having lichen than the CNN and dense neural network. Conversely, the CNN tended to predict no lichen (predicted value of zero) for more pixels that had lichen than either other model.

### 3.2 Test dataset blocks comparison

**Fig 9:**
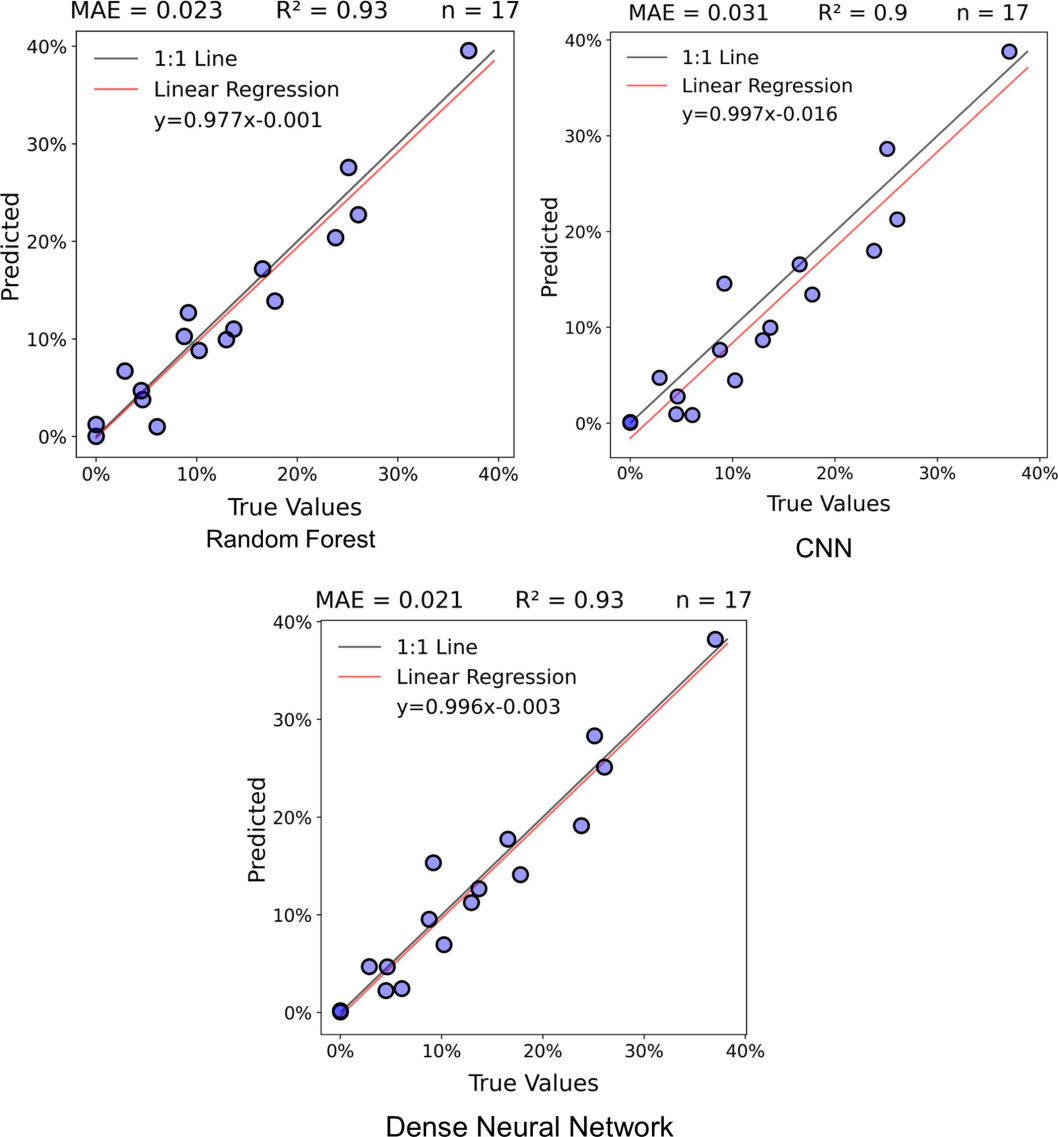
Graphic depicting mean block lichen percent coverage scatterplots, MAE, and R^2^ values for the models in this study.

This comparison assessed the performance of the models over larger areas, rather than comparing the individual pixels, by predicting the mean lichen percentage of each block within the test dataset. The test dataset consisted of 17 blocks from different sites, with a mean block size of 400 Sentinel-2 pixels. The dense neural network and random forest model had RMSE values of 0.027, while the CNN had a higher RMSE of 0.037. The random forest and dense neural network had an R^2^ of 0.93, showing a very high coefficient of determination (Fig 9). The MAE for the dense neural network and random forest were 2.1% and 2.3%, giving the dense neural network a slight edge. The CNN had a lower R^2^ (0.90) and a higher MAE (3.1%) than the other two models and showed a stronger systematic tendency to underpredict mean lichen percentage across blocks.

### 3.3 Lichen map using the dense neural network

**Fig 10:**
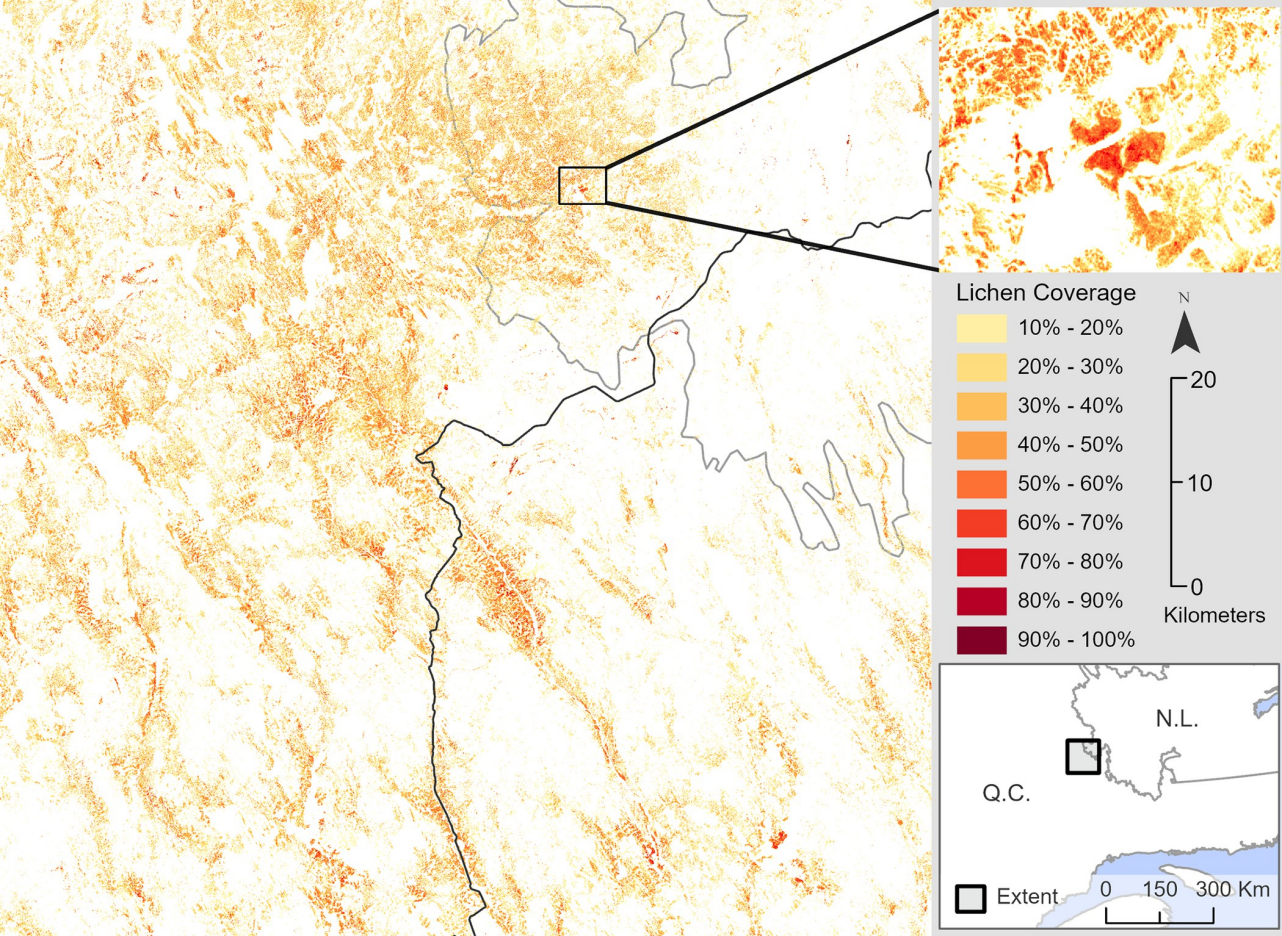
Lichen map of Fermont Quebec with inset of high lichen region. A lichen coverage of less than 10% is transparent in this map. Contains information licensed under the Open Government Licence–Canada.

We created a Sentinel-2 harmonized surface reflectance mosaic that covered most of the sites used in this study. The mosaic was created from the most recent cloud-free pixel from an August image. There were a few isolated instances, primarily over bodies of water, where the pixels had no imagery available. These pixels were filled in by using median focal statistics. The dense neural network model took 14 hours using an Intel i5-12600k CPU to create a 180 km^2^ regional lichen map (Fig 10).

### 3.4 Comparison of lichen map and true lichen values

**Fig 11:**
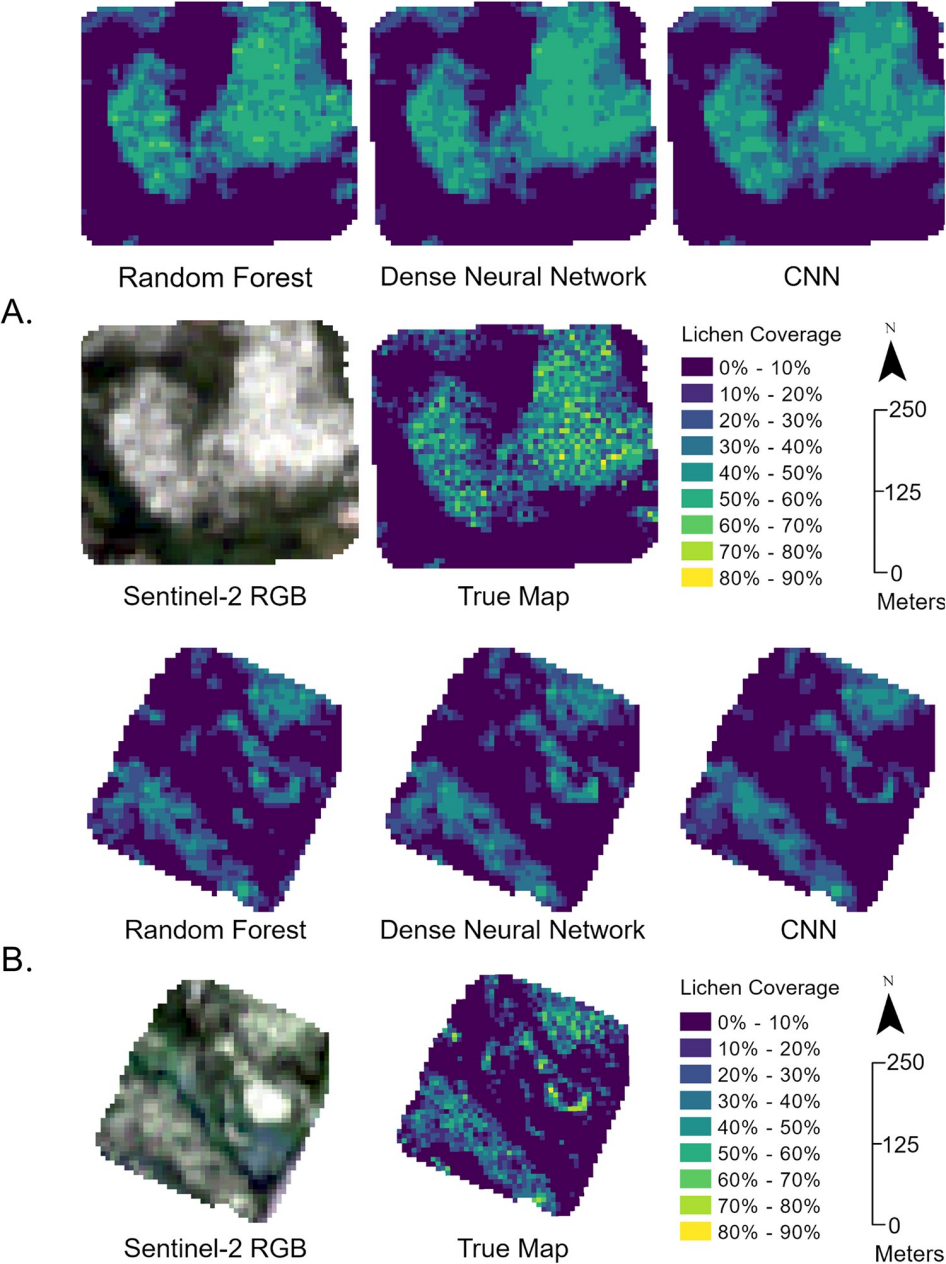
A. Comparison of lichen maps for Site C8, B. Comparison of lichen maps for site S4.

To better understand how the models performed at creating lichen maps, we compared the model generated lichen maps from the Sentinel-2 imagery mosaic with the true values for two of the 20 sites used in this study (C8 and S4). Site C8 consisted of very high lichen coverage interspersed between black spruce and Site S4 consisted of lichen interspersed between trees with a distinct parking lot surrounded by lichen. Fig 11 shows the random forest, dense neural network, CNN, and true lichen maps for these sites.

Across both sites, the predicted maps appear smoother and miss local variability found in the true lichen maps. This could be due to the spatial resolution of the Sentinel-2 sensor compared to the UAV imagery which was used to create the 10-m lichen maps. As observed in the scatterplot comparisons (Figs 8 and 9), the CNN had a greater tendency to underpredict lichen coverage percentage in these sites than the dense neural network and random forest model. This was apparent in the pattern of the lichen values in site C8, and the lower lichen values in the lichen pixels across site S4. The random forest model and the dense neural network produced similar lichen maps for site C8, with the random forest predicting more lichen pixels in the 60–70% bin than both the dense neural network and CNN.

**Fig 12:**
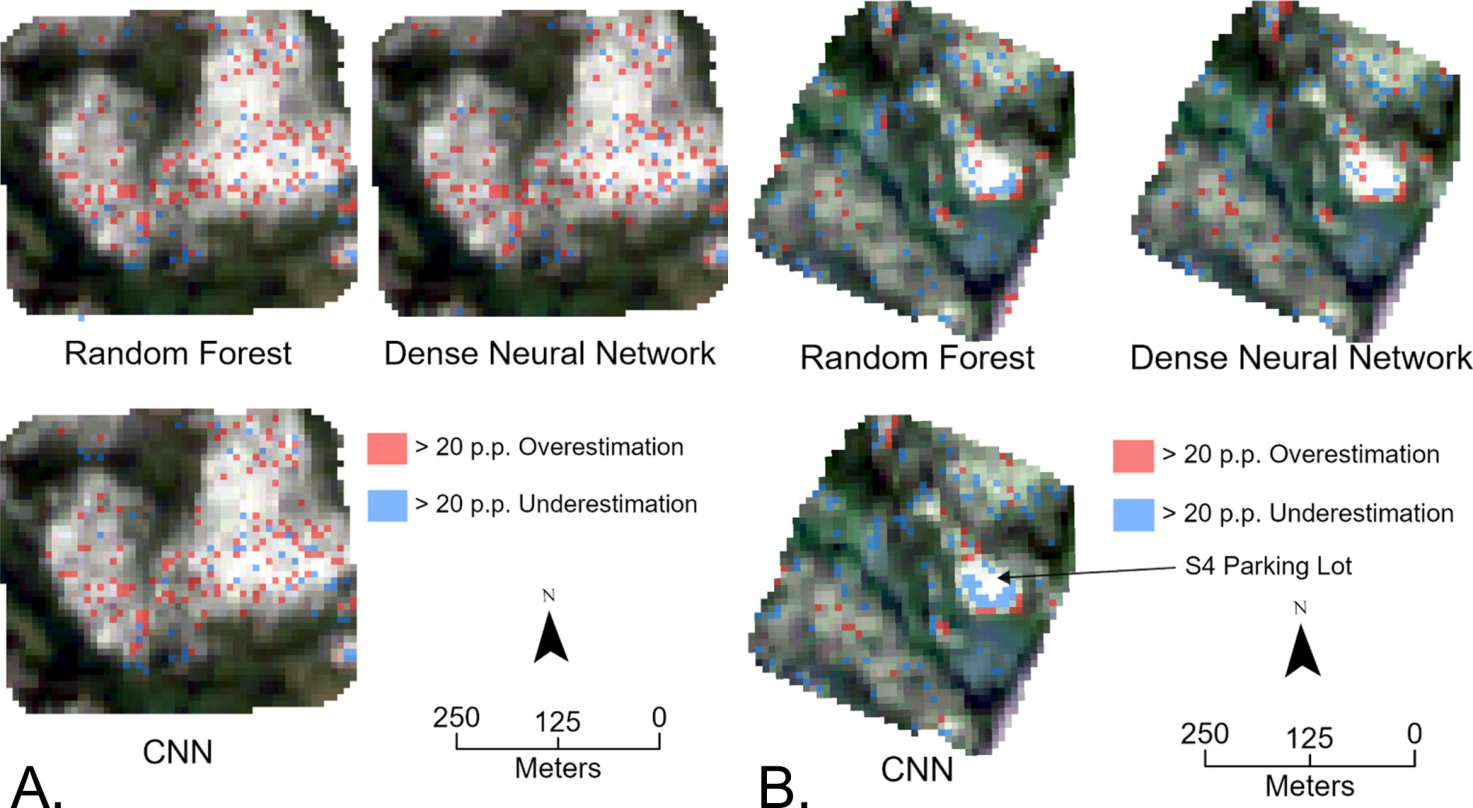
A. Map of overestimations and underestimations in site C8, B. Map overestimations and underestimations in site S4 with an arrow pointing to the parking lot.

We created difference maps between the true lichen maps and the predicted lichen maps to identify locations with large prediction errors. The difference map for both sites had mean errors of approximately 0 with standard deviations of approximately 10 percentage points (p.p.) difference between the predicted and true values. To highlight pixels that show substantial overestimation or underestimation, we thresholded each difference map at +/- 20 p.p. difference. Fig 12 shows the resulting difference maps where red and blue pixels represent substantial overestimates and underestimates respectively.

When evaluating site C8, all machine learning models had R^2^ values of 0.90, with the CNN having a slightly lower RMSE of 0.97 compared to the similar random forest and dense neural network RMSE of 0.098. Across all models, site C8 has a significant number of overestimations in the high lichen patches. This could be due to the difference in local variability found in the true lichen map and model predictions. When evaluating site S4, all models had a similar RMSE of 0.11, while the CNN had a lower R^2^ value of 0.78 compared to the 0.80 R^2^ from the random forest and dense neural network. The CNN systematically underestimates lichen in site S4, a pattern that is not replicated by the random forest and dense neural network. There are noticeable differences in model predictions of the site S4 parking lot surrounded with lichen, a feature consisting of high lichen coverage near exposed ground. Here, all models have numerous substantial overestimations and underestimations, with the CNN and random forest models having more underestimations than the dense neural network. Unlike the random forest model, neither the dense neural network nor the CNN had substantial misclassifications of the road in site S4.

## 4. Discussion

In this study, we compared three different types of machine learning models on their ability to predict lichen percent coverage from Sentinel-2 imagery. The output lichen coverage (%) maps from the 10-m Sentinel-2 imagery are created from freely accessible imagery and are higher resolution than Landsat lichen maps. Each type of model had different predictive performance, complexity, and computing power required for training. The dense neural network model outperformed the random forest and convolutional neural network models in terms of lower MAE in both test dataset comparisons, and a higher R^2^ in the test pixel comparison.

The dense neural network was significantly faster to train and did not require as many pre-processing steps as the CNN. A substantial challenge to training CNNs using TensorFlow is the reading of nine-band imagery. While opening three 16-bit PNG files was faster than opening one 9-band TIFF, the limiting factor in CNN training speed was the opening of files, which could potentially be resolved by accessing all the data from memory or using TFRecord files.

Herein, the most common hyperparameters for each model were tuned using grid search, with the sole purpose of finding the combination of parameters that minimized loss and maximized performance. Doing so was a valid attempt to undertake model tuning for the random forest, dense neural network, and CNN using the most common hyperparameters that are known to affect performance. While no claims are made that all models were tuned perfectly, all models were at the least optimized. The optimization provides a good degree of fairness among the three models that were developed.

A natural question that arises from this work is why the CNN did not perform better given the consensus that CNNs generally outperform dense neural networks and random forest models for image analysis problems. Often very deep CNNs such as VGG19, ResNet, and Xception are used in image analysis with a transfer learning approach, the most common being a pre-trained network with ImageNet weights [47]. Given the nature of the training data in this research, there were no well-proven CNN architectures or benchmark datasets that were considered sufficient to represent the problem at hand. Instead, we trained all models from scratch, with random weights initially assigned and used minimally deep networks for training.

We found that increasing the window size of the CNN tiles did not reduce the MAE for lichen mapping. Larger window sizes had a stronger tendency to overfit than smaller window sizes. The MAE on the test pixel dataset for the 7 px and 5 px tile models was 5.5%, while the best 3 px tile model had an MAE of 5.3% lichen coverage. Larger tile sizes could increase the noise introduced into the model and potentially lower the accuracy of predictions. Different types of CNNs were tested on all tile sizes, with some excluding dense layers and other models passing the central pixel values along with the flattened CNN feature map into dense layers. Overall, the CNNs that performed the best had a series of CNN layers, followed by a series of dense layers.

The dense neural network was applied to a Sentinel-2 surface reflectance harmonized mosaic to create the lichen map shown in Fig 10. A small number of pixels located inside mines, along route 389, or in the middle of lakes were predicted to have lichen coverage or NoData values. The models were not exposed to pixels with a reflectance similar to these land covers in the training since this study used lichen maps from vegetation surveys. Regional lichen maps could be improved by integrating land covers not found in UAV vegetation surveys into the datasets and either retaining the model or leveraging transfer learning, a neural network method of refining an existing model with new variables [21, 48].

There are significant limitations to the Sentinel-2 surface reflectance harmonized image collection found in GEE at the time of publishing, with no imagery located over our area of interest earlier than 2019. This complicates the process of creating lichen maps from earlier Sentinel-2 imagery or conducting regional time series analysis. However, the regional lichen maps generated in this study could be used as an intermediate step for training a Landsat-based lichen model that could be used to detect broader changes in lichen coverage over long periods.

The Sentinel-2 scale lichen maps created in this study could be useful for understanding current regional lichen coverage (%) in Quebec and Labrador Canada. The methodology of generating lichen percent coverage (%) maps, rather than classes of lichen coverage, could be used for quantifying the changes in lichen in a given region. This could be useful for tracking the amount of lichen available for caribou, contributing to caribou habitat mapping models, or determining how much lichen is lost from a given anthropogenic or natural cause.

## 5. Conclusion

The objective of this study was to determine whether UAV lichen maps could serve as training data for a machine learning model that predicts lichen in Sentinel-2 imagery of Quebec and Labrador Canada, and how different machine learning models ranked in this task. All machine learning models assessed in this study showed high performance in predicting lichen (%) coverage in Sentinel-2 imagery. For lichen mapping in this region, the dense neural network outperformed the CNN and random forest models. While the dense neural network requires more computational effort to train than a random forest model, the 5.9% performance gain in the test pixel comparison, and 9.1% performance gain in the quadrant block comparison renders it the most suitable for lichen mapping. The dense neural network trained on Sentinel-2 imagery had a pixel comparison MAE of 5.2% and R^2^ of 0.76, and a block comparison MAE of 2.1% and R^2^ of 0.93, making it appropriate for lichen mapping. Further research on retraining or transfer learning with the dense neural network is necessary for a regional lichen map to account for land covers not seen during the vegetation surveys used as training data in this study, such as mines and deep lakes. The Sentinel-2 lichen maps can be used for detailed caribou food source mapping, quantifying more recent changes in lichen coverage, or as training data for a model to predict lichen in Landsat imagery. This study represents substantial progress toward determining the appropriate methodology for generating accurate lichen maps to be used in caribou conservation and sustainable land management.

## Supporting information

S1 File The latest version of code for this paper, neural network model weights, Quebec and Labrador dense neural network lichen map, and training data can be accessed through GitHub: https://github.com/galenrichardson/Lichensen2modelcompare/, accessed on July 28, 2023. (DOCX)

S1 Appendix (DOCX)
