# Supplementary material for: Dense neural network outperforms other machine learning models for scaling-up lichen cover maps in Eastern Canada
Source: PLoS One. 2023 Nov 20;18(11):e0292839. doi: 10.1371/journal.pone.0292839 (PMC10659193; doi:10.1371/journal.pone.0292839)
Supplement: S1 File — (DOCX) [file pone.0292839.s001.docx]

**Supporting information**

The latest version of code for this paper, neural network model weights, Quebec and Labrador dense neural network lichen map, and training data can be accessed through GitHub: https://github.com/galenrichardson/Lichensen2modelcompare/, accessed on July 28, 2023.
