# Supplementary material for: Dense neural network outperforms other machine learning models for scaling-up lichen cover maps in Eastern Canada
Source: PLoS One. 2023 Nov 20;18(11):e0292839. doi: 10.1371/journal.pone.0292839 (PMC10659193; doi:10.1371/journal.pone.0292839)
Supplement: S1 Appendix — (DOCX) [file pone.0292839.s002.docx]

**Appendix A.**

We tested rectified linear unit (ReLU), exponential linear unit, linear, tangent, and sigmoid activation functions to see how they affected the dense neural network and CNN. Each activation function was tested with 16 different models before we determined that the ReLU activation function outperformed the other activation functions when used in all layers except for the last layer. A linear activation function was selected for the last layer to produce a continuous real output [1, 2]. We then tested the Adadelta, Adagrad, Adam, Adamax, Ftrl, Nadam, RMSprop, and Stochastic Gradient Descent optimizers by evaluating them on 16 different CNNs and dense neural networks. The Adam optimizer consistently outperformed other optimizers when integrated into CNNs and dense neural networks. We assessed different learning rate decay schedulers since learning rate decay has been found to improve the learning of complex problems while suppressing the memorization of noisy data [3]. We tested constant learning rate, exponentially decreasing, and step decreasing learning rate schedules on over different 100 CNNs and 100 dense neural networks. A model consisting of a constant learning rate performed best for dense neural networks and CNNs. While we did not train all the possible model shapes with the different activation functions, loss functions, optimizers, and learning rate decay schedulers, we deemed this process sufficient for understanding which kind of model performs well for this application.

**References**

1. Knudby A, Richardson G. Incorporation of neighborhood information improves performance of SDB models. Remote Sensing Applications: Society and Environment. 2023 Jul 20;101033.

2. Aptoula E, Ariman S. Chlorophyll-a Retrieval From Sentinel-2 Images Using Convolutional Neural Network Regression. IEEE Geosci Remote Sensing Lett. 2022;19:1–5.

3. You K, Long M, Wang J, Jordan MI. How Does Learning Rate Decay Help Modern Neural Networks? [Internet]. arXiv; 2019 [cited 2022 Nov 18]. Available from: http://arxiv.org/abs/1908.01878
